# Youth Needs at Intake into Trauma‐Informed Group Homes and Response to Services: An Examination of Trauma Exposure, Symptoms, and Clinical Impression

**DOI:** 10.1002/ajcp.12364

**Published:** 2019-08-01

**Authors:** Patrick M. Tyler, Irina Patwardan, Jay L. Ringle, Mary B. Chmelka, W. Alex Mason

**Affiliations:** ^1^ Child and Family Translational Research Center Boys Town NE USA

**Keywords:** Children and adolescents, Trauma‐informed care, Assessment and treatment, Residential care, Group homes

## Abstract

Trauma‐informed care is recommended to improve the quality of group home services for youth. Youth exposure to trauma and associated symptoms are important factors involved in making the clinical impression that determines treatment services. This study considered three dimensions of trauma (exposure, symptoms, and clinical impression) to determine associations with behavioral incidents of youth in trauma‐informed group homes and how trauma was related to changes in psychopathology from intake to discharge. Archival records of youth (*N *=* *1,096), age 9–18 (*M *=* *15.7 years) who received services from January 2013 to December 2017, and departed the program were used. Statistical procedures included hierarchical linear modeling and analysis of covariance. Results indicated trauma symptoms predicted emotional problems and self‐injurious behavior. Youth in high‐ and low‐trauma groups both showed decreases in behavioral incidents and psychopathology, but clinical impression of trauma moderated the change in emotional problems from intake to discharge. Youth deemed by clinicians to have lower trauma (based on history of maltreatment and expression of trauma symptoms) had greater decrease in emotional problems from admission to discharge. Limitations and implications for further research on implementation and effectiveness of trauma‐informed models are discussed.

## Highlights

- Trauma symptoms were related to emotional problems & self‐injurious incidents.
- High and low trauma groups both showed decreases in behavioral incidents and psychopathology.
- Youth deemed by clinicians to have lower trauma had greater decrease in emotional problems.
- Trauma symptoms and exposure should both be assessed when determining services.

## Introduction

Youth who receive services in group homes commonly have histories of exposure to trauma. The prevalence of experiencing multiple traumatic events such as abuse, neglect and violence can be as high as 92% for youth in residential care (Briggs et al., 2012). Unfortunately, it has been suggested that poorer outcomes are attributed to ineffective program models that may unintentionally use punitive practices that are similar to the child's earlier adverse experiences (Mohr, Martin, Olson, Pumariega, & Branca, 2009), resulting in coercive interactions between youth and staff (Gillen, 2012). To better serve youth across the United States, trauma‐informed services (U.S. Department of Health and Human Services [DHHS], 2014) are now a defined component for qualified residential treatment programs (QRTPs) in the Family First Prevention Services Act (FFPSA; Bipartisan Budget Act, 2018). Trauma‐informed QRTPs may reduce the likelihood of vulnerable youth experiencing further trauma during care, while facilitating responsiveness to services; however, studies examining the treatment needs and progress of youth within the context of established trauma‐informed group homes are lacking.

### Assessing Treatment Needs Associated with Trauma

Trauma should not be viewed as a unidimensional construct when making a clinical determination for treatment services. For example, clinicians might assess for exposure to traumatic events, without addressing the symptoms related to traumatic experiences (Rivard, Bloom, McCorkle, & Abramovitz, 2005). Thus, trauma refers not only to exposure to traumatic events or various adverse childhood experiences (ACEs; Felitti et al., 1998), but also to trauma symptomatology and the resulting degree of clinical impairment experienced. For a full representation of this complex construct, studies are needed that examine all three dimensions (exposure, symptoms, and clinical impression of impairment) simultaneously; yet, many studies have addressed only one in isolation. Importantly, these dimensions map onto considerations in the delivery of trauma‐informed residential care placements such as group homes. Such care starts with trauma screening and assessments to identify whether a youth has experienced a traumatic event and, if so, has reactions to the event (National Child Traumatic Stress Network, 2018). This information informs the clinical impression used to determine service planning objectives and strategies for addressing targeted outcomes (Bright, Raghavan, Kliethermes, Juedemann, & Dunn, 2010). Little research has examined how the combination of trauma exposure, symptoms, and clinical impression may differentially predict youth treatment needs and response to group home services.

### Trauma‐Informed Services

Applying a trauma‐informed approach in a residential setting *“*presumes every child has likely been exposed to abuse, neglect, or other traumatic experiences” (Association for Children's Residential Centers, 2014, p. 99). Because the degree of trauma exposure and symptoms can vary in youth in residential programs, general organizational characteristics and program components have been identified to facilitate trauma‐responsive care. One study reported the following organizational factors were related to successful implementation of trauma‐informed procedures and settings: physical and emotional safety, trustworthiness, youth choice and control, collaboration and empowerment, and youth and caregiver involvement in discharge and transition planning to the next placement (Hummer, Dollard, Robst, & Armstrong, 2010). Additionally, specific practices recommended for trauma‐informed services include addressing trauma symptoms, preventing recurrence of trauma, psychoeducation, and identification and management of trauma‐related triggers (DHHS, 2014).

Studies that have examined how trauma can impact youth response to treatment services have been mostly conducted in residential treatment centers that are more restrictive settings than group homes. For example, Boyer, Hallion, Hammell, and Button (2009) found the number of trauma experiences youth had was inversely correlated with the likelihood of improvement based on clinical level changes in psychopathology. Other studies have evaluated youth response to programs that used trauma‐informed or trauma‐focused approaches. Hodgdon, Kinniburgh, Gabowitz, Blaustein, and Spinazzola (2013) showed psychopathology decreased in girls who received residential treatment services using a trauma‐informed model. A quasi‐experimental study by Rivard et al. (2005) found youth who received residential treatment services that addressed post‐traumatic stress symptoms, using a trauma recovery framework, demonstrated more improvement in pro‐social and self‐reflective problem‐solving compared to youth who received standard services. A randomized controlled trial that tested Trauma‐Focused Cognitive Behavior Therapy showed significant improvement in PTSD and depressive symptoms with youth who had different types and severity of trauma (Cohen et al., 2016). Minimal research, however, is available on how trauma impacts youth response to trauma‐informed group homes services.

### Youth in Group Homes

Group homes are service settings that assist youth with behavior modification, skill development, and crisis intervention from trained staff (Scott & Lorenc, 2007), while also providing other services such as mental health and substance abuse counseling, education, psychiatry, and health care (Lieberman, 2004). Youth can be referred to group homes for a variety of issues that can include academic problems, emotional and behavioral disorders, drug and alcohol dependency, and delinquency (Connor, Miller, Cunningham, & Melloni, 2002; Farmer, Dorsey, & Mustillo, 2004; Larzelere, Daly, Davis, Chmelka, & Handwerk, 2004; Lyons & Schaefer, 2000; Trout et al., 2008). Pane, Farmer, Wagner, Maultsy, and Burns (2015) reported three‐quarters of youth in group homes had a history of neglect, one‐third had physical and/or sexual abuse, and one‐fifth had experienced emotional abuse. Although exposure to traumatic experiences, such as childhood maltreatment, may not be a reason a youth is referred to a group home, trauma exposure can impact the level of severity of their clinical presentation such as suicidality (Briggs et al., 2012), psychiatric symptoms (MacDonald et al., 2016), and physical health (Felitti et al., 1998). The type of maltreatment may also impact youth differently. For example, prior work has shown emotional abuse and sexual abuse, compared to emotional and physical neglect, were significantly more positively associated with trauma symptoms and emotional problems (Tyler et al., 2019). This is especially common in girls who experience higher rates of sexual abuse victimization (Martinez, Polo, & Zelic, 2014; Mueser & Taub, 2008) and report higher trauma symptoms than boys (American Psychiatric Association [APA], 2013; Martinez et al., 2014; Steinberg et al., 2013). Youth, therefore, may have different responses to group home services as a function of their varying experiences of and reactions to trauma, including traumatic stress symptoms (Kisiel, Summersett‐Ringgold, Weil, & McClelland, 2017). The importance of considering both trauma symptoms and type of maltreatment in determining the services needed to address youth clinical needs has, therefore, been recommended (Pane et al., 2015).

The purpose of this study was to evaluate how three dimensions of trauma (exposure, symptoms, and clinical impression) are associated with youth disruptive behavior, self‐injurious incidents, and psychopathology (i.e., conduct and emotional problems) at intake and during trauma‐informed services, and to determine whether these trauma dimensions moderate the changes made during youths’ stay. Gender was also included in the analysis based on the notable differences between girls and boys in the experiences and consequences of trauma (APA, 2013; Martinez et al., 2014; Mueser & Taub, 2008; Steinberg et al., 2013). Three research questions were addressed as follows: (a) Are trauma exposure, trauma symptoms, clinical impression, and gender differentially associated with behavioral incidents, such as disruptive and self‐injurious behaviors, and psychopathology at the beginning of services? (b) Does trauma exposure, trauma symptoms, clinical impression, or gender moderate the change in behavioral incidents (i.e., disruptive behavior and self‐injurious incidents) from month to month during care? (c) Does trauma exposure, trauma symptoms, clinical impression, or gender moderate the change in psychopathology (i.e., conduct and emotional problems) from intake to discharge? We expected that youth with exposure to a greater number of traumatic events, elevated trauma symptoms, and, particularly, significant impairment as determined by clinical impression would present with higher levels of disruptive behavior and self‐injurious incidents at intake. Moreover, in this study's trauma‐informed setting, we expected that the three dimensions of trauma would not moderate response to care, suggesting that all youth would make similar improvements (i.e., have parallel slopes capturing changes in behavior and psychopathology, despite having different intercepts or starting points), but that gender differences in response to care would be observed. This study was conducted to help practitioners and researchers determine factors related to the treatment needs of youth that have been impacted by trauma. Additionally, this study provided preliminary evidence on youth treatment response to a trauma‐informed group home model that can be used in future program improvement and evaluation studies, including randomized controlled trials.

## Method

### Participants and Procedures

The sample consisted of 1,096 youth, ages 9–18 (*M *=* *15.7 years; *SD *=* *1.6), from a large agency that provides group home services in the Midwest. Archival records of youth who received services from January 2013 to December 2017 and departed the program were used for the analysis. Sixty‐six percent were male, and 44% were Caucasian, 27% were African American, 13% were Hispanic, 11% were more than one race, 4% were American Indian, and 1% were Asian. The sample had a median length of stay of 308 days with a range 8–1,559 days. The agency's Institutional Review Board approved the procedures for the study.

### Setting and Program Description

All of the youth who participated in the study had resided and received trauma‐informed group home services in the Boys Town Family Home Program (Father Flanagan's Boys Home [FFBH], 2015), which implements a modified version (Thompson & Daly, 2015) of the evidence‐based Teaching‐Family Model (TFM; California Evidence‐Based Clearinghouse, 2018; James, 2011; Wolf, Kirigin, Fixsen, Blasé, & Braukmann, 1995). TFM group homes are the most widely practiced treatment model for group homes (Farmer et al., 2004). Professionally trained staff called Family‐Teachers reside with the youth in a family‐style environment. They teach youth pro‐social skills, relationship building, motivation skills, self‐government, problem‐solving, and moral/spiritual development (FFBH, 2015; Kirigin, 2001; Thompson & Daly, 2015; Wolf et al., 1995). Agencies that use the TFM have certification standards that require monitoring the “prevalence of trauma and how trauma affects all individuals involved with the program” (Teaching‐Family Association, 2018). Trauma‐informed components of the model include staff training to identify and understand the types and effects of trauma, and the importance of providing a calm and nurturing environment that ensures youth are physically and emotionally safe. Strategies include teaching and reinforcing skills for youth and their families with praise and encouragement that promote self‐advocacy, empowerment, conflict resolution, healthy decision‐making, coping and stress management skills, self‐control, and regulation strategies. In addition, the model teaches youth how to express feelings and stop negative thoughts, as well as relaxation strategies to help youth cope with the symptoms related to traumatic stress such as anxiety (Dowd & Tierney, 1992). The frequency and duration of strategies are prescribed in the youth's individualized service plan based on intake assessments, youth and family input, staff observations, documentation of behavioral incidents, and clinical impression.

### Measures

Information from youth records and intake assessments was used to operationalize three predictors (trauma exposure, trauma symptoms, and clinical impression of impairment based on trauma) and two outcome measures (behavioral incidents while in the program and level of psychopathology at intake and discharge). Cut‐scores for trauma exposure, symptoms, and clinical impression were calculated to aid in the application of findings to practice. Finally, measures are a combination of mean scales and counts. Alpha reliabilities are reported for mean scales only as internal consistency was not expected in the case of counts and thus not reported.

#### Predictors

##### Trauma exposure

Exposure was measured according to nine constructs adapted from the Adverse Childhood Experiences study (ACE; Felitti et al., 1998). Archival data, based on administrative records and structured checklists gathered by admissions counselors from caregivers and youth, were the sources of the exposure variables. This included three types of abuse (emotional, physical, and sexual) and one global type of neglect. In addition, five other types of exposures were defined: partner violence, household substance use, household mental health issues, parent relationship problems, and criminal household member. Items were dichotomized (0 = *no*, 1 = *yes*), and youth could receive scores ranging from 0 to 9.

##### Trauma symptoms

Symptoms were assessed with the Brief Trauma Symptom Scale for Youth (BTSSY; Tyler et al., 2019), which was adapted from the Primary Care–Post‐Traumatic Stress Disorder Screen (PC‐PTSD; Prins et al., 2003) for the use with children and adolescents. The BTSSY consists of six items (e.g., intrusive thoughts, physiological reactions) based on the Diagnostic and Statistical Manual of Mental Disorders—5 (American Psychiatric Association, 2013) symptoms of post‐traumatic stress disorder (PTSD). Youth completed a self‐report paper version of the BTSSY, which was administered by program staff during the Youth Program Orientation following admission. Youth were asked to respond to each statement on a 3‐point Likert‐type scale ranging from 0 (*not true*) to 2 (*certainly true*). Symptoms were calculated as a sum of the six symptom scores and had an internal consistency of α = .77. Prior testing showed the instrument had acceptable reliability and validity for screening PTSD to determine the need for additional assessment (Tyler et al., 2019).

##### Clinical impression of impairment based on trauma

Service Planning Assessment Tool (SPAT; FFBH, 2012) ratings, completed by program clinicians, were used for the clinical impression of impairment based on trauma. Two items of the SPAT were used by clinicians to determine goals, objectives, and strategies related to trauma for the youth and family service plan. The first item was expression of trauma and was based on dysregulation of emotions, fears, triggers, anxiety, sexualized behaviors, and acting‐out behaviors. The second SPAT item was history of maltreatment and was based on history of physical, sexual, emotional abuse, neglect, and abandonment. Indicators are rated on a five‐point (0–4) Likert scale (0—*No evidence*, 1—*Present but does not affect daily living*, 4—*Negatively affects every aspect of child's life*). For example, a score of four would be defined as “negatively affects every aspect of the child's life, e.g., has extreme behaviors related to trauma/maltreatment, cannot function well due to thoughts/feelings about trauma/maltreatment.” Expression of trauma and history of maltreatment scores from the SPAT were combined into one clinical impression score with an internal consistency of α = .87. Although practitioners used trauma exposure and symptom information to formulate clinical impressions, the SPAT scores included their clinical judgment to determine how trauma impacted the clinical needs of the youth to determine services. The clinical impression score, therefore, provided a third perspective that was in addition to the sheer number of adverse childhood experiences and youth self‐report of trauma symptoms.

#### Outcomes

##### Behavioral incidents

Youth behavioral incidents were reported by the Family‐Teachers using the Daily Incident Report (DIR; Handwerk et al., 2006). The DIR consisted of incident report data collected from the agency's electronic youth record based on staff observations of significant youth behaviors that were documented daily. Report information included the date, time, and description of the event, and was coded by the supervisor according to established definitions based on the incident type. Definitions for the incidents were acquired from the training manual for the program. For example, *physical aggression* was defined as “Program participant engages in physically aggressive behaviors such as throwing objects, slamming doors, overturning furniture, or slamming fists.” All of these significant incidents required immediate response and reporting by Family‐Teachers to their direct supervisor per agency policy. For example, any indication of aggressive behavior or suicidal ideation by youth was reported by Family‐Teachers to their supervisor who completed a safety assessment with the youth.

The reliability of the DIR has been established in several studies. First, Wright (2001) investigated how likely Family‐Teachers were to report youths’ problem behaviors to clinical supervisors. Using a questionnaire distributed to 54 Family‐Teachers containing 43 scenarios, reporting reliability for all events was 83.5%, indicating a moderate but acceptable level of agreement between Family‐Teachers and clinical supervisors. Additionally, Larzelere (1996) conducted analyses of inter‐coder reliability of the narratives by administrative staff. Kappa coefficients ranged from .66 to .97 (*M *=* *.91) for codes entered for the same narratives by different coders. Therefore, at the level of coding the narrative descriptions, the DIR possesses good to excellent reliability. Taken together, both at the level of reporting and coding, the DIR appears to possess acceptable reliability.

For this study, fifteen indicators of behavioral incidents were aggregated into two indices capturing disruptive behavioral incidents (12 items) and self‐injurious incidents (three items). Disruptive behavioral incidents included behaviors such as physical aggression and property damage. Self‐injurious incidents included self‐destructive behavior, suicidal ideation, or suicide attempt. Because the daily incident data are zero inflated, and to minimize the number of data points in the longitudinal analysis, the sum of disruptive and, separately, the sum of self‐injurious behaviors per month were calculated for each youth over their first year in the program. As such, each youth potentially could have up to 12 disruptive and self‐injurious behavior scores.

##### Psychopathology

Youth psychopathology was assessed with the conduct and emotional problems subscales of the Strengths and Difficulties Questionnaire (SDQ; Goodman, Meltzer, & Bailey, 1998) that has acceptable reliability and validity as a brief measure of psychopathology in children and adolescents (Goodman, 2001). The SDQ is a 25‐item questionnaire designed to assess child behavioral and emotional problems. The Conduct Problems subscale consists of five items related to child conduct problems (e.g., “often fights with other children or bullies them”), and the Emotional Problems subscale consists of five items related to child's emotional problems (e.g., “often seems worried”). Conduct and emotional problems were rated by the caregivers at intake and by Family‐Teachers at discharge. Each rater indicated how true each statement described youth's behavior on 3‐point Likert‐type scale ranging from 0 (*not true*) to 2 (*certainly true*).

### Data Analyses

Secondary data analysis was conducted on archival records of clinical data on youth who received services from January 2013 to December 2017 and departed the program. First, the prevalence of trauma exposure, symptoms, and clinical impression, disruptive behavior and self‐injurious incidents, and psychopathology (conduct and emotional problems) were examined for the full sample, and for girls and boys separately. Descriptive analyses were conducted using SPSS 25 (IBM, 2017). To examine gender differences in the prevalence of traumatic dimensions and targeted outcomes, chi‐square tests for dichotomous outcomes and t‐tests for continuous outcomes were conducted. Pearson product–moment correlation coefficients were computed to assess bivariate associations among trauma, behavioral incidents, and psychopathology outcomes. Steiger's *z*‐tests were used to compare the difference between two correlations with common variables (Steiger, 1980).

To test whether trauma exposure, symptoms, and clinical impression of trauma moderated change in psychopathology and behavioral incidents over time, we conducted two types of analyses. The associations of trauma dimensions with changes in behavioral incidents during youth's stay in the Family Home Program were analyzed using hierarchical linear modeling (HLM6; Raudenbush & Bryk, 2002) and conducted using time in care in months. Level 1 variables (i.e., within‐subject variables) included dummy‐coded trauma factors (high vs. low), youth age at admission, and gender as Level 2 variables (i.e., between subjects variables). To test whether the different types of trauma and youth gender differentially predicted change in youth psychopathology from intake to discharge, a 2 × 2 × 2 × 2 four‐way repeated‐measures analysis of covariance was conducted, with four independent predictors (exposure, symptoms, clinical impression, and gender), while controlling for youth age at admission. Prior to analysis, each trauma variable was split into high and low groups based on cutoff procedures described below, and girls were coded lower than boys (0 = *girl*, 1 = *boy*).

## Results

Table 1 displays the means and standard deviations of the three trauma dimensions and their indicators for the total sample as well as by gender. In terms of trauma exposure, a cut‐score of ≥5 was determined, based on prior research (Bruskas & Tessin, 2013; Hodgdon et al., 2013), with a *z*‐score of .53 which identified 70% in the low‐trauma group for exposure. Specifically, more than 50% of the total sample indicated that they were exposed to poor parental anger control, substance abuse in the family, and parent marital discord. Girls reported higher levels of physical (35% vs. 25%), χ^*2*^(1, *N *=* *1084) = 11.91, *p *=* *.001, and sexual abuse (38% vs. 8%), χ^2^(1, *N *=* *1083) = 147.05, *p *<* *.001, mental illness in the family (45% vs. 31%), χ^2^(1*, N = *1084) = 21.41, *p *<* *.001, having a criminal parent (46% vs. 36%), χ^2^(1, *N *=* *1084) = 9.25, *p *=* *.003, and neglect (64% vs. 55%), χ^2^ (1, *N *=* *1084) = 20.95, *p *<* *.001. For trauma symptoms, a cut‐score of ≥3 was used (see Prins et al., 2003, 2016; Tyler et al., 2019) and identified 62% in the low‐trauma group. Girls also had significantly higher total trauma symptoms compared to boys *t*(980) = 7.85, *p *<* *.001. In terms of clinical impression, a cut‐score of ≥4 was determined based on a *z*‐score of .54 which identified 71% in the low‐trauma group. Girls also scored significantly higher than boys on expression of trauma *t*(896) = 10.35, *p *<* *.001 and history of maltreatment *t*(869) = 8.79, *p *<* *.001.

**Table 1:** Trauma exposure, symptoms, clinical impression: girls and boys at intake

	Total		Girls		Boys		*p*
	*M*	*SD*	*M*	*SD*	*M*	*SD*	
Trauma exposure^a^	3.81	2.24	4.42	2.41	3.50	2.09	***
Poor anger control (parent)	0.61	0.49	0.66	0.48	0.59	0.49	*
Physical abuse	0.28	0.45	0.35	0.48	0.25	0.43	**
Sexual abuse	0.18	0.39	0.38	0.49	0.08	0.27	***
Partner violence	0.24	0.43	0.27	0.45	0.22	0.42	
Substance abuse (family)	0.59	0.49	0.62	0.49	0.57	0.50	
Mental illness (family)	0.35	0.48	0.45	0.50	0.31	0.46	***
Parental discord	0.58	0.49	0.60	0.49	0.57	0.50	
Criminal parent	0.39	0.49	0.46	0.50	0.36	0.48	**
Neglect	0.58	0.49	0.64	0.48	0.55	0.50	***
Trauma symptoms^b^	2.24	2.48	3.08	2.78	1.81	2.18	***
Avoid situations	0.66	0.76	0.91	0.78	0.54	0.71	***
Numb/disconnected	0.24	0.49	0.33	0.53	0.20	0.46	***
Easily startled	0.54	0.67	0.64	0.68	0.49	0.66	**
Intrusive thoughts	0.27	0.55	0.38	0.63	0.22	0.50	***
Distressing dreams	0.28	0.58	0.42	0.69	0.20	0.49	***
Physiological reactions	0.25	0.54	0.41	0.65	0.17	0.45	***
Clinical impression^c^	2.72	2.39	3.71	2.29	2.08	2.23	***
History of maltreatment	1.44	1.32	1.91	1.29	1.14	1.25	***
Expression of trauma	1.30	1.21	1.80	1.15	0.99	1.13	***

^a^ *n *= 1,085.

^b^ *n *= 980.

^c^ *n *= 843.

*p <* *.05. **p <* *.01 .***p <* *.001.

Means and standard deviations of the psychopathology and behavioral incident variables are shown for the total sample and by gender in Table 2. Girls had higher emotional problems at intake, *t*(1029) = 6.93, *p *<* *.001, whereas boys had higher conduct problems, *t*(1029) = −2.67, *p *=* *.008. Girls, on average, also had higher self‐injurious behaviors *t*(682.807) = 3.31, *p *=* *.001, during their stay, whereas girls and boys did not differ on disruptive behaviors, *t*(1088.584) = −1.75, *p *=* *.08. Overall, females demonstrated significantly more self‐injurious behaviors during their time in care and were experiencing significantly more emotional problems. Boys on the other hand were reported to have significantly more conduct problems at intake.

**Table 2:** Psychopathology and behavioral incidents: comparison of girls and boys

	Total		Girls		Boys		*p*
	*M*	*SD*	*M*	*SD*	*M*	*SD*	
SDQ intake total (caregiver report)^a^	16.00	6.94	16.03	6.79	15.98	7.02	
Conduct problems	4.30	2.63	4.01	2.68	4.45	2.59	*
Emotional problems	3.46	2.42	4.16	2.39	3.09	2.36	***
Daily incident report^b^							
Disruptive behaviors	0.79	2.05	0.66	1.15	0.85	2.38	
Self‐injurious behaviors	0.06	0.22	0.09	0.23	0.04	0.21	**

SDQ= Strength and Difficulties Questionnaire.

^a^ *n *= 1,017.

^b^ *n *= 1,096.

*p <* *.05. **p <* *.01. ***p <* *.001.

Correlations between the trauma factors, behavioral incidents, psychopathology, and youth age at admission are displayed in Table 3. There were significant positive correlations among the three trauma factors, with a significantly larger association (Steiger's *z* = 5.38, *p *<* *.001) between exposure and clinical impression (*r *=* *.48, *p *<* *.001) compared to symptoms and clinical impression (*r *=* *.28. *p *<* *.001). There was also a small negative correlation (*r *=* *−.07) between exposure and clinical impression with age (*p *<* *.05), indicating higher scores for younger youth. All three trauma dimensions had a positive association with emotional problems at intake; however, the relationship was significantly stronger between emotional problems and trauma symptoms compared to the other two factors (Steiger's zs = 3.30–3.72, *p *<* *.01). Symptom was the only trauma factor that was significantly associated with self‐injurious behaviors (*r *=* *.15, *p *<* *.001). None of the trauma dimensions were correlated with disruptive behaviors or conduct problems at intake. As a result, the positive correlation between all three trauma factors and emotional problems was significantly greater compared to the relationship with conduct problems (Steiger's *z* = 3.78–7.39, *p *<* *.001).

**Table 3:** Correlations of trauma factors, behavioral incidents, psychopathology, and age

Variable	1	2	3	4	5	6	7	8
1. Trauma exposure								
2. Trauma symptoms	.26^a^							
3. Clinical impression	.48^a^	.28^a^						
4. Disruptive behaviors	.03	.04	.03					
5. Self‐injurious behaviors	.03	.15^a^	.03	.48^a^				
6. Conduct problems	−.02	−.02	−.02	.12^a^	.03			
7. Emotional problems	.12^a^	.26^a^	.12^a^	−.02	.10^a^	.26^a^		
8. Age	−.07^a^	.04	−.07^a^	−.20^a^	−.11^a^	−.16^a^	−.03	

a *p *<* *.05.

### HLM Analysis of Trauma and Disruptive and Self‐Injurious Behavioral Incidents

Given that the incident report variables were count data, a Poisson sampling model with a log link function was used (Raudenbush & Bryk, 2002). Preliminary examination indicated that the data would fit better if it was winsorized to the 85th percentile. Winsorizing is a method of dealing with outliers by making all extreme scores equal (e.g., values in the 85th percentile) in order to pull in outliers.

#### Disruptive behaviors

The unconditional model revealed that disruptive behaviors were significant, indicating that there was significant variation in this outcome variable (β = −0.196), *t*(738) = −4.43, *p *<* *.001. The level one model indicated that the initial deceleration (square root of time) in disruptive behaviors was non‐significant, (β = 0.096), *t*(738) = 1.00, *p *=* *.07, whereas the overall time trend was significant (β = 0.086), *t*(738) = −5.31, *p *<* *.001. The three trauma dimensions were added to the model as well as age at admission (grand mean centered at 15.7 years) and gender as Level 2 predictors. None of the trauma dimensions nor gender were significantly associated with the intercept, reflecting behavioral incidents in the first month of the program. However, age at admission was significant (β = −0.173, *p < *.001), indicating that younger children engaged in more disruptive behaviors during the first month of their stay. For the time components, all variables were non‐significant, indicating that all youth changed at the same rate over time. These analyses are not reported in full but are available on request.

#### Self‐injurious behaviors

**Figure 1:**
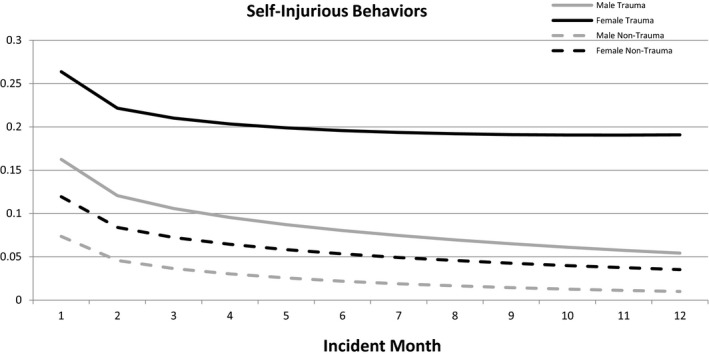
Example of estimated self‐injurious behaviors over time for boys versus girls with and without reported trauma at admission

Self‐injurious behaviors were significant for the unconditional base model, indicating that there was significant variation in the measured behaviors (β = −0.61), *t*(738) = −32.88, *p *<* *.001. The level one model indicated that the initial deceleration (square root of time) in self‐injurious behaviors was significant, (β = −0.364), *t*(738) = −2.93, *p *=* *.004, whereas the overall time trend was non‐significant (β = −0.001), *t*(738) = −.02, *p *=* *.985. The three trauma predictors were added into this model as well as age at admission (grand mean centered at 15.7 years) and gender (0 = girl, 1 = boy) as Level 2 predictors. Table 4 presents the fixed‐effects, event rate ratios, and 95% confidence intervals for this analysis. In the first month of the program (the intercept), those entering the program with high trauma symptoms engaged in significantly more self‐injurious behaviors than those with low symptoms (β = 0.778, *p < *.001). The same pattern occurred with high clinical impression (β = 0.466, *p < *.05). Conversely, and unexpectedly, those who experienced high trauma exposure engaged in significantly fewer self‐injurious behaviors than those with low exposure (β = −0.451, *p < *.05). Finally, during the first month in the program, boys engaged in significantly fewer self‐injurious behaviors than girls (β = −0.484, *p < *.05). Age at admission was not a significant predictor of self‐injurious behaviors during the first month (β = −0.103, *p = *.113). Auxiliary statistics indicated that having the Level 2 variables on the intercept accounted for 35% of the variance in self‐injurious behaviors, whereas the full model accounted for 40% of the variance, an increase of 5%. Figure 1 displays fitted lines for four different combinations of trauma and gender. Girls and those youth who had higher trauma symptoms tended to have more self‐injurious behaviors during their first month in care. However, the figure indicates parallel slopes over time suggesting that all groups’ rates of self‐injurious behaviors decreased at a similar rate.

**Table 4:** Log‐linear model for self‐injurious behaviors

Variable	β	*SE*	Event rate ratio	95% CI	*p*
Intercept (self‐injurious behaviors)	−2.125	.26	0.12	0.07, 0.20	***
Mean age at admission	−0.103	.06	0.90	0.80, 1.02	
Trauma exposure	−0.451	.19	0.63	0.44, 0.93	*
Trauma symptoms	0.778	.20	2.17	1.46, 3.25	***
Clinical impression of trauma	0.466	.21	1.59	1.06, 2.40	*
Male	−0.484	.21	0.62	0.41, 0.93	*
Month in program (slope)	−0.007	.10	0.99	0.82, 1.21	
Mean age at admission	0.001	.03	1.00	0.95, 1.06	
Trauma exposure	−0.008	.09	0.99	0.83, 1.19	
Trauma symptoms	0.052	.09	1.05	0.89, 1.25	
Clinical impression of trauma	−0.004	.10	1.00	0.82, 1.22	
Male	−0.048	.09	0.95	0.79, 1.14	
Month in program^2^ (slope)	−0.344	.32	0.71	0.38, 1.32	
Mean age at admission	−0.089	.08	0.91	0.79, 1.07	
Trauma exposure	0.269	.27	1.31	0.77, 2.23	
Trauma symptoms	−0.117	.26	0.89	0.53, 1.50	
Clinical impression of trauma	−0.016	.30	0.98	0.55, 1.76	
Male	−0.075	.28	0.93	0.54, 1.60	

CI = Confidence Interval.

*df* = 733.

* *p* < .05.

*** *p* < .001.

### Trauma and Psychopathology

#### Conduct problems

A repeated‐measures four‐way ANCOVA determined that the number of conduct problems at intake (*M *=* *4.23, *SE *=* *.14) was higher than at discharge (*M *=* *3.84, *SE *=* *.15), *F*(1, 501) = 4.94, *p *=* *.027. The effect of trauma was non‐significant regardless of the trauma measure, indicating that youth in the high and low levels of trauma groups changed the same. A test of between‐subject effects revealed a significant main effect of age *F*(1, 501) = 21.44, *p *<* *.001, and gender *F*(1, 501) = 12.57, *p *<* *.001. More specifically, follow‐up pair‐wise comparisons indicated that, on average, girls (*M *=* *3.62, *SE *=* *.17) had fewer conduct problems compared to boys (*M *=* *4.42, *SE *=* *.15), *F*(1, 501) = 12.57, *p *<* *.001, and younger youth had more conduct problems (*r *=* *−.16, *p* < .001) than older youth.

#### Emotional problems

**Figure 2:**
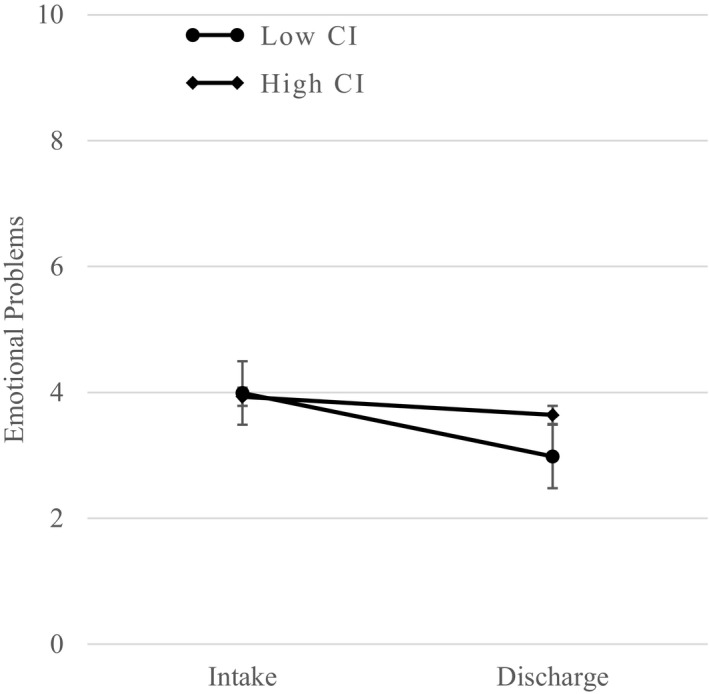
Average decline in emotional problems for youth with low versus high clinical impression of trauma. Error bars represent standard errors. CI = clinical impression.

A repeated‐measures four‐way ANCOVA determined that the number of emotional problems at intake (*M *=* *3.96, *SE *=* *.12) was higher than at discharge (*M *=* *3.31, *SE *=* *.14), *F*(1, 501) = 16.41, *p *<* *.001. Tests of between‐subject effects revealed a significant main effect of gender *F*(1, 501) = 23.31, *p *<* *.001, and a significant main effect of trauma symptoms, *F*(1, 501) = 27.84, *p *<* *.001. Follow‐up pair‐wise comparisons indicated that, on average, girls (*M *=* *4.13 *SE *=* *.13) had more emotional problems than boys (*M *=* *3.15, *SE *=* *.13), and youth in the low‐trauma symptom group as measured by the BTSSY had less emotional problems (*M *=* *3.10, *SE *=* *.13) than youth in the high trauma symptom group (*M *=* *4.17, *SE *=* *.15). Tests of within‐subject effects revealed a significant time by clinical impression interaction (*p *=* *.03), as shown in Fig. 2. Post hoc simple effect analyses indicated that the decrease in emotional problems was significant for the group with low trauma based on clinical impression, *F*(1, 500) = 17.86, *p *<* *.001, but not in the group with high clinical impression *F*(1, 500) = 1.82, *p *=* *.18. At time 1, there was not a significant difference in mean levels of emotional problems between high and low clinical impression groups, but these levels were significantly different at time 2. The low clinical impression group had a significant mean decrease from intake to discharge (*M *=* *3.98 vs. *M *=* *2.99), while the high clinical impression group showed a decrease but it was non‐significant (*M *=* *3.92 vs. *M *=* *3.63).

## Discussion

In this study, we examined how trauma exposure, symptoms, clinical impression of trauma, and gender were related to behavioral incidents, psychopathology, and subsequent changes in youth receiving services in trauma‐informed group homes. Unlike many studies that have examined a single trauma dimension in isolation, we examined exposure, symptoms, and clinical impression to determine whether these dimensions differentially predicted disruptive and self‐injurious behavioral incidents or psychopathology (i.e., conduct problems and emotional problems). Specifically, analyses were conducted to determine whether each dimension of trauma or gender moderated changes in behavioral incidents and, separately, psychopathology during services. It has been suggested that residential placement can reproduce the adverse conditions of trauma exposure for vulnerable youth, thereby exacerbating trauma symptoms and inhibiting treatment gains (Gillen, 2012; Mohr et al., 2009). By contrast, trauma‐informed services, a requirement set forth for QRTPs in the recently passed FFPSA (Bipartisan Budget Act of 2018, 2018), may address youths’ needs in a trauma‐responsive manner and facilitate improvements during their out‐of‐home stays. We conducted three sets of analyses to address these expectations.

For our first research question, we analyzed correlations to determine the degree to which the three trauma dimensions were associated with one another and differentially associated with behavioral incidents and psychopathology at the beginning of services. Results revealed that there was a stronger positive correlation between exposure and clinical impression compared to symptoms, which may be an indication that clinicians relied more heavily on exposure to make their clinical impression of impairment. Still, the correlations among trauma dimensions were not so high as to suggest they capture the same information, suggesting the possibility of differential associations with other constructs. Although relationships between the three trauma dimensions and disruptive behaviors or conduct problems were not evident, all three trauma dimensions were associated with self‐injurious behaviors in the first month of care and with emotional problems at intake. Trauma symptoms were significantly more positively associated with self‐injurious incidents and emotional problems, and the only dimension, along with gender, that was significant when all trauma dimensions were included in the ANCOVA for emotional problems. Similarly, Leenarts et al. (2013) found symptoms of PTSD predicted mental health problems among girls in residential care. The difference in positive association between trauma symptoms with self‐injurious behaviors and emotional problems compared to externalizing incidents and conduct problems also converges with prior research (Leenarts et al., 2013; Tyler et al., 2019; Yoon, Steigerwald, Holmes, & Perzynski, 2016) and suggests that trauma symptoms of youth in group homes may be more likely to manifest in negative affect and self‐injurious behaviors than in disruptive behaviors.

For the second research question, analyses of behavioral incidents showed that exposure had an inverse relationship with self‐injurious behaviors, indicating that higher exposed youth had significantly fewer self‐injurious behaviors in the first month. This was an unexpected finding given prior research showing an inverse relationship between trauma exposure and improvement in treatment based on changes in psychopathology (Boyer et al., 2009). The negative association between trauma exposure and self‐injurious behavior, in our sample, could be a result of inconsistencies in information reported, which has been indicated by Leenarts et al. (2013). For example, studies on abuse reporting indicate minimization of abuse can be as high as 30%–40% (MacDonald et al., 2016) and inconsistency in reports of abuse can be due to reasons such as the sensitivity of the topic, avoidance, embarrassment, or difficulty remembering information about traumatic events (McKinney, Harris, & Caetano, 2009). Youth and family members receiving residential services may be especially hesitant to share information related to trauma until rapport and trust can be established with service providers.

Results further indicated that youth with high trauma symptoms and clinical impression had higher rates of self‐injurious behaviors when controlling for age and gender. Girls also had significantly higher self‐injurious behavior than boys, like prior results (Handwerk et al., 2006). Self‐injurious behaviors, however, decreased over time for both groups, and this change was not moderated by either the trauma dimensions or gender. Disruptive behaviors were higher for younger youth in the first month, but there was no difference for trauma and gender in the first month nor did the amount of change over time differ based on gender or trauma. The overall decrease in behavioral incidents in the entire sample replicated earlier results from Handwerk et al. (2006) and the decreased levels of psychopathology were similar to work by Hodgdon et al. (2013) who showed psychopathology decreased in girls receiving residential treatment services that used a trauma‐informed model. Our study extended prior research by examining the potential differences that exposure, symptoms, and clinical impression had on response to services in a large sample of boys and girls in group homes. It is also important to note, there was no evidence of potential iatrogenic effects regarding behavioral incidents and psychopathology for vulnerable youth during their stay.

Finally, for the third research question, our analyses of psychopathology indicated conduct problems were higher at intake compared to discharge for all groups of youth. Gender was a significant predictor, with higher conduct problems at intake for boys compared to girls. This was slightly different than prior research by Handwerk et al. (2006), which found girls had higher emotional and conduct problems compared to boys at intake. However, there were no significant interactions for conduct problems, which indicated that the decrease from intake to discharge was not moderated by trauma or gender. It should be noted that trauma exposure was positively correlated with emotional problems, in contrast to the inverse relationship found with self‐injurious behavioral incidents. Youth with high trauma on all three trauma dimensions also had higher emotional problems compared to youth with low trauma when tested independently, and girls had higher levels than boys. Notably, youth in both groups showed a positive response to services as evidenced by a decrease in conduct and emotional problems. Trauma symptoms and gender were the only significant predictors of emotional problems when controlling for the other variables in the model, but change over time was moderated by clinical impression of impairment.

Of the three trauma dimensions, only clinical impression moderated change from intake to discharge. Clinical impression was based on the service planning ratings of impairment due to traumatic symptoms and history of maltreatment determined by clinical staff according to the information gathered from the youth, caregivers, and youth intake records. The pattern of this statistical interaction indicated that emotional problems were higher for the low clinical impression compared to the high group at intake. This could be an indication that some youth had higher emotional problems regardless of trauma or that the reported information was inconsistent. This study could not determine whether clinicians were consistently using the trauma exposure information or the trauma symptoms to determine the clinical impression. Given the pattern of correlations, it is possible that clinicians rely more on exposure rather than symptoms to make their clinical impression as suggested by Rivard et al., 2005. If so, then increased use of trauma symptom information may provide improved insight into the emotional problems of youth.

In future studies, we will determine the practical significance of this result, since clinical impression scores are used by program staff to determine service plan objectives and interventions for the youth. For example, a mixed‐methods analysis could be conducted to determine how trauma exposure and symptom information are used in making the clinical impression. Service plan data (i.e., goals, objectives, and strategies) could also be used to investigate the alignment of trauma‐specific strategies to the clinical impression, which could further provide an indication of youth response to individualized trauma‐specific interventions. The organization also routinely monitors program implementation by conducting observations of staff with instruments that measure the quality of model fidelity. Data from model fidelity observations could be used to assess implementation of trauma‐informed practices to determine quality assurance as well as the relationship between these practices and youth response to services.

There are several strengths to this study, including the focus on multiple dimensions of trauma, the large sample size, and the real‐world practice setting. There also are some noteworthy limitations. The study was based on archival data collected originally for clinical purposes in a single agency. The trauma measures for exposure and clinical impression require further psychometric testing. In addition, the BTSSY was only collected at intake, which prevented the analysis of change in trauma symptoms from intake to discharge. The degree to which results can be generalized to other programs or settings is also uncertain. Finally, this study was correlational; therefore, future research is needed to experimentally or quasi‐experimentally test the effectiveness of the Family Home Program before any causal interpretations can be made.

In conclusion, the context of residential care is changing across the United States considering passage of the FFPSA (Bipartisan Budget Act of 2018, 2018), which requires QRTPs to offer trauma‐informed care. However, research on this topic is limited. The current study sought to address this gap. First, the positive treatment response demonstrated by youth overall to this trauma‐informed group home model was promising, but further analysis is needed to identify the specific components of the model that can help youth with trauma further decrease their emotional problems. Second, further inquiry is needed to understand how information related to trauma exposure and symptoms is used to make the clinical determination for treatment services. For instance, relying solely on trauma exposure for the clinical impression might miss youth with treatment needs related to trauma because of underreporting. Overreliance on trauma exposure may also, consequently, result in underutilizing information youth may share related to trauma symptoms. Viewing both trauma exposure and symptoms as two‐sides‐of‐the‐same‐coin is therefore recommended to improve the clinical impression and judgment needed to determine treatment services. Ongoing collaboration between researchers and practitioners should therefore continue to identify and test the effectiveness of trauma‐informed models, as well as the assessments and strategies that can help youth with high levels of trauma achieve the same outcomes as their peers during and after care.

## Conflict of Interest

There were no disclosures of conflict of interest.
